# Improved detection of local tumor progression after ablation or resection of colorectal liver metastases using [^18^F]FDG-PET/contrast-enhanced CT versus contrast-enhanced CT alone: results from a Dutch prospective cohort

**DOI:** 10.1007/s00330-026-12726-x

**Published:** 2026-07-11

**Authors:** O. D. Bijlstra, S. van Mossel, B. Boekestijn, M. C. Burgmans, E. L. van Persijn van Meerten, D. D. D. Rietbergen, S. Shahbazi Feshtali, R. J. Swijnenburg, F. H. P. van Velden, H. Koffijberg, L. F. de Geus-Oei, J. S. D. Mieog

**Affiliations:** 1 Department of Surgery, Leiden University Medical Center, Leiden, The Netherlands; 2 Department of Surgery, Amsterdam University Medical Center, Amsterdam Cancer Center, Amsterdam, The Netherlands; 3 Department of Radiology, Leiden University Medical Center, Leiden, The Netherlands; 4 Health Technology and Services Research Department, Technical Medical Centre, University of Twente, Enschede, The Netherlands; 5 Biomedical Photonic Imaging Group, University of Twente, Enschede, The Netherlands; 6 Department of Radiation Science and Technology, Delft University of Technology, Delft, The Netherlands

**Keywords:** Colorectal liver metastases, Follow-up, Local treatment, [^18^F]FDG, Ablation

## Abstract

**Objective:**

Post-treatment surveillance after locally treated colorectal liver metastases involves CEA measurement and contrast-enhanced CT (CECT) scans. However, reactive tissue around ablated lesions can appear similar to viable tumor tissue on CECT, complicating the detection of local tumor progression (LTP). Therefore, a new imaging protocol was initiated, which incorporates [^18^F]FDG-PET/CT to complement CECT. This study evaluated the diagnostic accuracy of [^18^F]FDG-PET/CECT for early detection of disease progression after resection or thermal ablation, compared to CECT alone.

**Materials and methods:**

The study analyzed a prospective cohort of patients undergoing thermal ablation or resection, who received CECT and [^18^F]FDG-PET/CT scans 3–5 months post-treatment. Disease progression was confirmed by histology, consensus during a multidisciplinary team meeting or after extended follow-up. Diagnostic accuracy of [^18^F]FDG-PET/CECT was compared to standard practice (CEA serum levels and CECT).

**Results:**

The final analysis included 64 patients with 154 lesions. 37 patients underwent ablation, 14 patients underwent resection, and 13 patients underwent combination therapy, resulting in 93 (60%) lesions being treated with thermal ablation and 61 (40%) lesions with resection. Disease progression was found in 66% of patients, with LTP detected in 25% of treated lesions. Compared to CECT, [^18^F]FDG-PET/CECT had a higher diagnostic accuracy for detecting local progression after thermal ablation (AUC 0.97 vs. 0.73, *p* = 0.001) and hepatic resection (AUC 0.96 vs. 0.69, *p* = 0.03), and for detection of extrahepatic disease (AUC 0.91 vs. 0.79, *p* = 0.03). CEA serum levels had low diagnostic accuracy for detecting disease progression (AUC 0.60).

**Conclusion:**

[^18^F]FDG-PET/CECT improves diagnostic accuracy in the early follow-up of patients with locally treated colorectal liver metastases.

**Key Points:**

***Question****: Interpreting CECT scans after local treatment of colorectal liver metastases can be challenging because reactive tissue around treated lesions can appear similar to viable tumor tissue*.

***Findings****: [*^*18*^*F]FDG-PET/CECT improves the diagnostic accuracy of detecting local tumor progression after thermal ablation and resection of colorectal liver metastases*.

***Clinical relevance****: [*^*18*^*F]FDG-PET/CECT should be considered during follow-up after local treatment of colorectal liver metastases to identify and treat local tumor progression earlier*.

**Graphical Abstract:**

**Figure:**
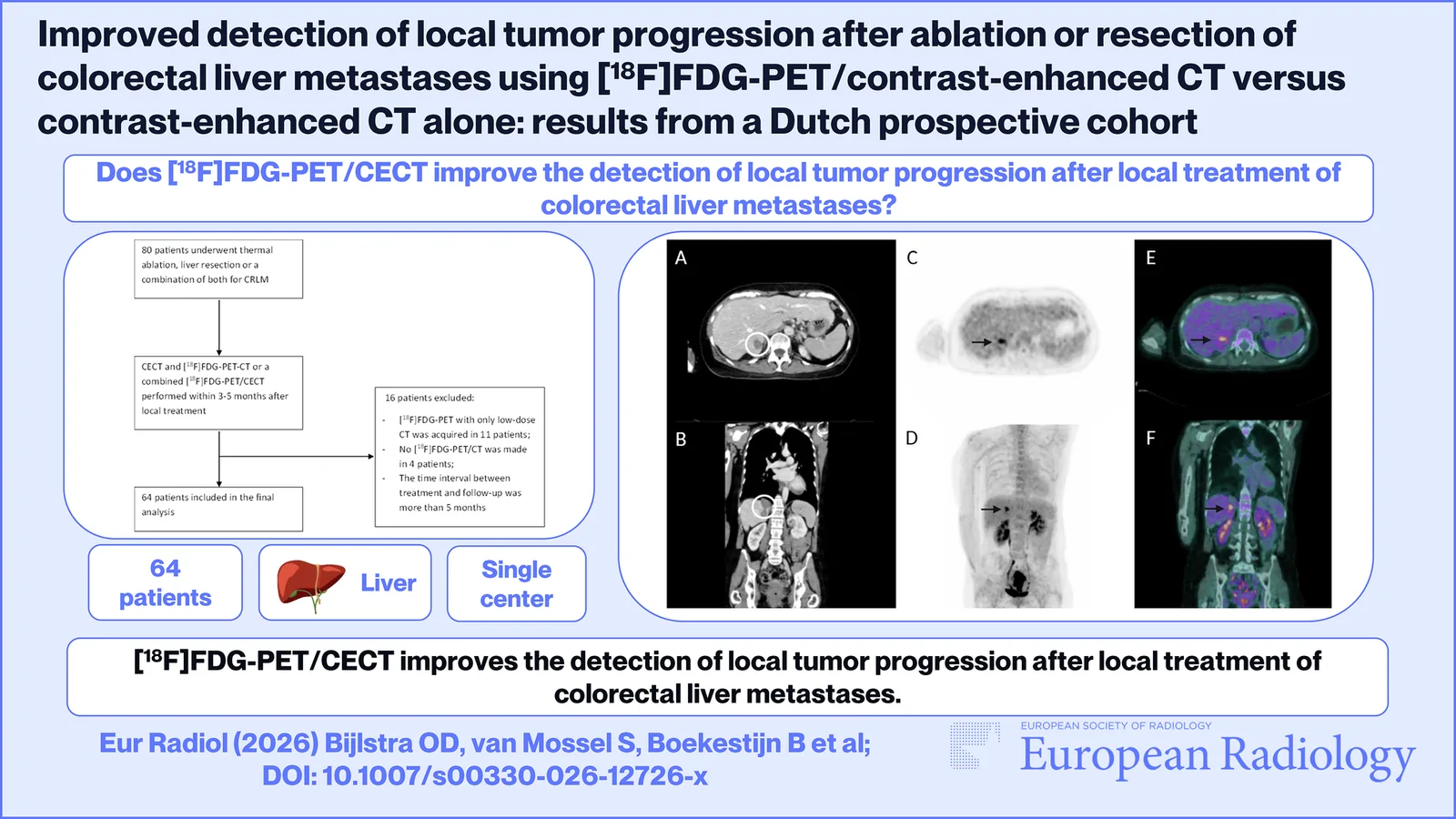

## Introduction

Colorectal cancer (CRC) is the most prevalent gastrointestinal malignancy worldwide, with the liver being the most common site of metastases, affecting approximately 30%–50% of patients [1–3]. Hepatic resection and thermal ablation (i.e., radiofrequency or microwave ablation (RFA, MWA)) are currently the only curative treatment options. The probability of subsequent disease-free survival strongly depends on the disease stage, tumor biology and treatment efficacy. In current clinical practice, morphological imaging with conventional contrast-enhanced CT (CECT), complemented by serum CEA, is the modality of choice for evaluating (local) tumor progression. Following local therapy, changes of liver tissue make evaluation by morphological imaging more challenging, resulting in false negative and false positive test results, which, on the one hand, can lead to treatment delay and avoidable disease progression, and on the other hand, to unnecessary diagnostics and/or futile treatment [4].

Adding [^18^F]FDG-PET/CT to CECT imaging combines both metabolic and morphological imaging. Complementary metabolic information enables the detection of malignant disease at unexpected sites or within morphologically normal structures that would otherwise be easily overlooked on CECT. There is increasing use of contrast-enhanced magnetic resonance imaging (MRI) to complement CECT in the diagnostic work-up of patients with colorectal liver metastases (CRLM). Recently, the CAMINO trial, an international, multicenter, prospective diagnostic-accuracy study, reported frequent changes in the local treatment plan based on liver MRI [5]. Although the value of MRI before local treatment has become clear, it, like CECT, only provides morphological imaging characteristics. The advantage of [^18^F]FDG-PET/CECT over CECT alone is mainly attributed to more accurate tumor detection by demonstrating focally increased glucose metabolism in liver tissue compared to strictly morphologic assessment. Purely structural tissue changes in the liver can be misleading due to postinterventional non-malignant changes, such as fibrosis or necrosis in the ablation or resection zone [6], and reactive tissue may present as a hypodense area around the ablated lesion, often making it indistinguishable from viable tumor tissue. A possible lower false negative rate of [^18^F]FDG-PET/CECT leads to earlier detection of local tumor progression (LTP), which may facilitate prompt locoregional treatment options.

The role of [^18^F]FDG-PET/CECT imaging in the diagnostic work-up and during follow-up after local treatment remains unclear in current national and international guidelines [3, 7–9]. In recent consensus guidelines by the European Society for Medical Oncology (ESMO), no recommendation regarding the use of [^18^F]FDG-PET/CECT after locoregional therapy was provided [9]. In line with the ESMO consensus guidelines, Martin et al conclude that the role of [^18^F]FDG-PET/CECT in the postoperative follow-up of patients with CRLM has not yet been defined [3], whereas the ECIO-ESOI consensus recommendations in 2020 state that [^18^F]FDG-PET/CECT is superior to CECT alone for detecting LTP [8].

The main aims of diagnostic follow-up after local treatment are early detection of residual tumor tissue, LTP, and new intrahepatic and extrahepatic metastases. Most frequently, disease recurrence occurs in the first three years after local treatment, and the prevalence peaks in the first 6 months [10]. Therefore, the addition of [^18^F]FDG-PET/CT to the early follow-up strategy after local treatment might increase diagnostic accuracy in order to treat patients earlier with curative intent to improve overall survival and quality of life. Our hospital’s protocol was changed prior to this study, and all patients undergoing either thermal ablation or liver resection received a [^18^F]FDG-PET/CECT at first follow-up imaging.

In this retrospective single-center cohort study, the diagnostic value of adding [^18^F]FDG-PET/CT to standard practice (i.e., CECT and serum CEA level) was studied in the follow-up of patients with CRLM who underwent thermal ablation, hepatic resection or both.

## Materials and methods

### Patients

A waiver was obtained by the division’s medical ethics review board to perform this study. Informed consent was obtained from all participants. Data were collected from all patients receiving local treatment, either thermal ablation or hepatic resection for CRLM, from 01-06-2020 until 31-12-2021 in a single university medical center in the Netherlands. Patients were included in the study if they underwent open or minimally invasive metastasectomy, radiofrequency—and/or MWA or a combination of these treatments, followed by a post-procedural abdominal CECT and follow-up [^18^F]FDG-PET/CECT 3–5 months after local treatment. When performing, the maximum interval between follow-up CECT and [18F]FDG-PET/CT separately was one week. Patients harboring liver metastases of a different origin than CRC were excluded from the analysis. A patient screening flowchart is displayed in Supplementary Fig. 1. Additional data—on the clinical condition of the patient, location of primary tumor and hepatic metastases characteristics, history of systemic therapy, Fong clinical risk score, and previous local hepatic treatments provided—was retrospectively extracted from the hospital’s electronic patient record.

### Local therapy

Percutaneous RFA and MWA procedures were performed under anesthesia using ultrasound (US) and/or CT-guidance for needle positioning, and a CECT was acquired directly after the ablation to assess the extent of the ablation zone. Open ablation was performed during surgery under general anesthesia, and US was used for needle positioning. For the RFA procedures, a multiple-probe Cooltip (Medtronic) was used and EMPRINT (Medtronic) for MWA procedures. The duration of ablation was determined at the discretion of the interventional radiologist and documented in the treatment report. Ablation procedures were performed by one of four experienced interventional radiologists, assisted by radiology technologists. Hepatic resection was performed by one of four experienced hepatobiliary surgeons, either by open or by minimally invasive parenchymal-sparing or anatomic resection, depending on the number and location of metastases.

### Follow-up

Patients treated with percutaneous RFA or MWA received a baseline CECT scan directly after ablation. In patients treated by a combination of resection and thermal ablation, a CECT scan was acquired before hospital discharge. The first follow-up scan was made 3–5 months after local treatment and was complemented with CEA serum levels. All but five patients received a top-to-mid-thigh [^18^F]FDG-PET/CECT scan; the remaining 5 patients received a [^18^F]FDG-PET/CECT scan of the liver only. Recurrence of disease was divided into three groups: local recurrence, new (non-local) CRLM and extrahepatic disease (EHD).

### Post-treatment image acquisition

[^18^F]FDG-PET/CT scans were acquired according to the EANM guidelines [11] using an EANM Research Ltd (EARL)-accredited PET/CT scanner (Vereos, Philips Healthcare). Patients fasted for 6 h prior to intravenous [^18^F]FDG injection and were pre-hydrated with 1 L of water in the 2 h prior to the scan [12]. Acquisition of PET images occurred 60 min (55–75 min) after [^18^F]FDG administration. [^18^F]FDG dose was calculated according to the quadratic formula (MBq): 379 × (patient weight (kg)/75)^2^/time per bed position (min·bed^−1^). For patients undergoing PET/CECT of the liver only, the acquisition time was 5 min·bed^−1^. For all other patients, the acquisition time was 2–4 min·bed^−1^ (depending on patient weight). Prior to the PET acquisition, patients underwent a low-dose CT scan for attenuation correction purposes (120 kVp, 35 mAeff). All PET data were corrected for decay, scatter, randoms and attenuation, and were reconstructed using an EARL ^18^F standards 1-compliant reconstruction protocol [13], i.e., blob-based iterative reconstruction algorithm (3 iterations, 15 subsets) followed by a 5.5 mm full-width-at-half maximum Gaussian filter, using an isotropic voxel size of 4 mm. After PET acquisition, a helical CECT of the abdomen was performed in the portal venous phase using 100 kVp, a gantry rotation time of 0.5 s, a large scan field of view, and a standard reconstruction kernel. The collimation was 0.5 × 80 mm. Iobitridol (Xenetix® 350 mg/mL) was administered as the contrast agent, injected over 30 s followed by a saline flush. Contrast dose was calculated based on the patient’s body weight (1.2 mL/kg).In some cases, the [^18^F]FDG-PET/CT and CECT were acquired on different days. In these cases, the CECT was performed with the Aquilion ONE (Canon Medical Systems Europe) using a closely matched acquisition protocol.

### Evaluation of post-treatment CECT and [^18^F]FDG-PET/CECT imaging

All [^18^F]FDG-PET/CECT scans were independently interpreted after anonymization by two experienced nuclear medicine physicians (L.F.G. and D.R.) in a random order using Sectra IDS7 (Sectra AB). [^18^F]FDG-PET/CECT scans were considered positive for LTP when increased focal FDG uptake was observed that exceeded the uptake of the adjacent healthy liver background and could not be attributed to post-ablation inflammatory response, which typically presents with a more diffuse rim-like FDG uptake pattern. New CRLM and EHD were also documented. CECT scans were anonymized and shown in a random order and were independently interpreted by two experienced abdominal radiologists (SSF and EPM) using Sectra IDS7. The abdominal radiologists were blinded to the PET/CT scans and the PET/CT reports. LTP was defined as any morphologically detectable activity inside or abutting the ablation zone or resection margin with or without enhancement of the rim.

### Reference standard

A grading system for the level of evidence of the reference standard was established. Recurrence confirmed by biopsy or liver resection was considered the highest level of evidence. However, because a biopsy was only obtained in one patient, histopathological confirmation could not serve as the reference standard. The second level of evidence consisted of the growth of suspicious lesions observed on follow-up imaging, with or without rising CEA levels. The follow-up period was three years. The lowest level of evidence was based on the multidisciplinary review board’s decision to perform re-ablation, based on available follow-up imaging and elevated pathological CEA levels.

### Statistical analysis

Lesion-based and patient-level observational outcomes were reported and analyzed. All findings were compared with the reference standards. Findings were classified as false-negative, false-positive, or true test results for CECT alone vs. combined [^18^F]FDG-PET/CECT to calculate the sensitivity, specificity, positive predictive value (PPV), and negative predictive value (NPV). Differences in outcomes and diagnostic accuracy between CECT and [^18^F]-FDG-PET/CECT were tested using McNemar’s test and the *χ*^2^ test. Receiver operating characteristic (ROC) curves and the area under the curve (AUC) were calculated using the ROC analysis under the paired-samples, based on the empirical methods described by DeLong et al [14]. Statistical significance was defined as a *p*-value less than 0.05. All data were collected and analyzed using IBM SPSS Version 25.0.

## Results

A total of 64 patients with 154 lesions were included in the final analysis. The median time between initial treatment and follow-up imaging was 122 days (interquartile range 113–131). Nine patients received a separate CECT and [^18^F]FDG-PET/CT. The median interval between CECT and [^18^F]FDG-PET/CT was 0 days (range 0–7 days). Thirty-seven patients were treated by thermal ablation only, 14 patients underwent hepatic resection only, and 13 patients were treated with a combination of both treatments, resulting in 93 (60%) lesions being treated by thermal ablation and 61 (40%) lesions by hepatic resection. Median age of patients was 64 years at the time of treatment, and all patients had primary colon or rectal cancer. Patients who underwent surgery were more likely to receive neoadjuvant chemotherapy (9/16 = 56% vs. 6/37 = 16%, *p* = 0.004), had larger tumors (mean lesion size 21 vs. 11 mm, *p* = 0.001) and more lesions (mean number of lesions 5.4 vs. 1.7, *p* < 0.001) than patients treated by thermal ablation. In the thermal ablation group, significantly more patients (23 vs. 3, *p* < 0.001) were treated for recurrent CRLM than in the liver metastasectomy group. Only in one patient, a biopsy was taken during follow-up before repeat local treatment, in 52 (81%) patients consensus during the MDT based on imaging and CEA levels was used as reference standard, whereas for 11 patients (17%) a wait-and-see policy was decided. In 16 (25%) patients an additional MRI scan of the liver was acquired because no consensus was reached during the MDT, confirming the suspicion of LTP and/or new CRLM in 15 patients. LTP was found in 16 (25%) patients, new CRLM in 16 (25%) patients, and new EHD in 22 (34%) patients on first follow-up imaging. Median disease-free survival was 125 days (interquartile range 118–577), and 21 (33%) patients were disease-free 1 year after local treatment, and 14 (22%) patients remained disease-free at 3-year follow-up. The reliability of the MDT consensus was confirmed in all (22) patients without disease progression at first follow-up and after 3 years of follow-up. Eighteen patients underwent thermal re-ablation of the liver or stereotactic radiotherapy of the lung directly after the first follow-up imaging. Three patients had suspicious lesions at first follow-up imaging, which were confirmed malignant during further follow-up imaging, and subsequently underwent thermal ablation of the liver or stereotactic radiotherapy of the lung. Nineteen patients were directly referred for palliative (chemotherapy) treatment after the first follow-up imaging. One patient was diagnosed with LTP by the MDT consensus and did not receive treatment. During further follow-up, the lesion disappeared. Baseline characteristics are summarized in Table 1.

**Table 1:** Baseline data of included patients according to treatment group

	Thermal ablation	Hepatic resection	Combined therapy	Cohort (*n*=64)	*p*-value
**Number of patients**	**37**	**14**	**13**	**64**	
Age (median)	65	62	61	64	0.74
Colon cancer (%)	23 (62)	11 (79)	8 (62)	42 (66)	0.51
Rectal cancer (%)	14 (38)	3 (21)	5 (38)	22 (34)	
Neoadjuvant chemotherapy (%)	6 (16)	9 (60)	4 (30)	19 (30)	0.004*
**Number of lesions**	**93**	**61**	**NA**	**154**	
Mean lesion size (mm)	11	21	NA		0.001*
Fong score:					0.79
Low (≤ 2)	19	7	8		
High (> 2)	18	7	5		
Recurrent CRLM (%)	24 (38)	3 (21)	1 (8)	28 (44)	< 0.001*
ASA score high (%)	15 (41)	2 (14)	3 (23)	20 (31)	0.15

^*^ Statistically significant *p*-values are bold

### Diagnostic accuracy of CECT and [^18^F]FDG-PET/CECT for the detection of LTP after thermal ablation

**Fig. 1:**
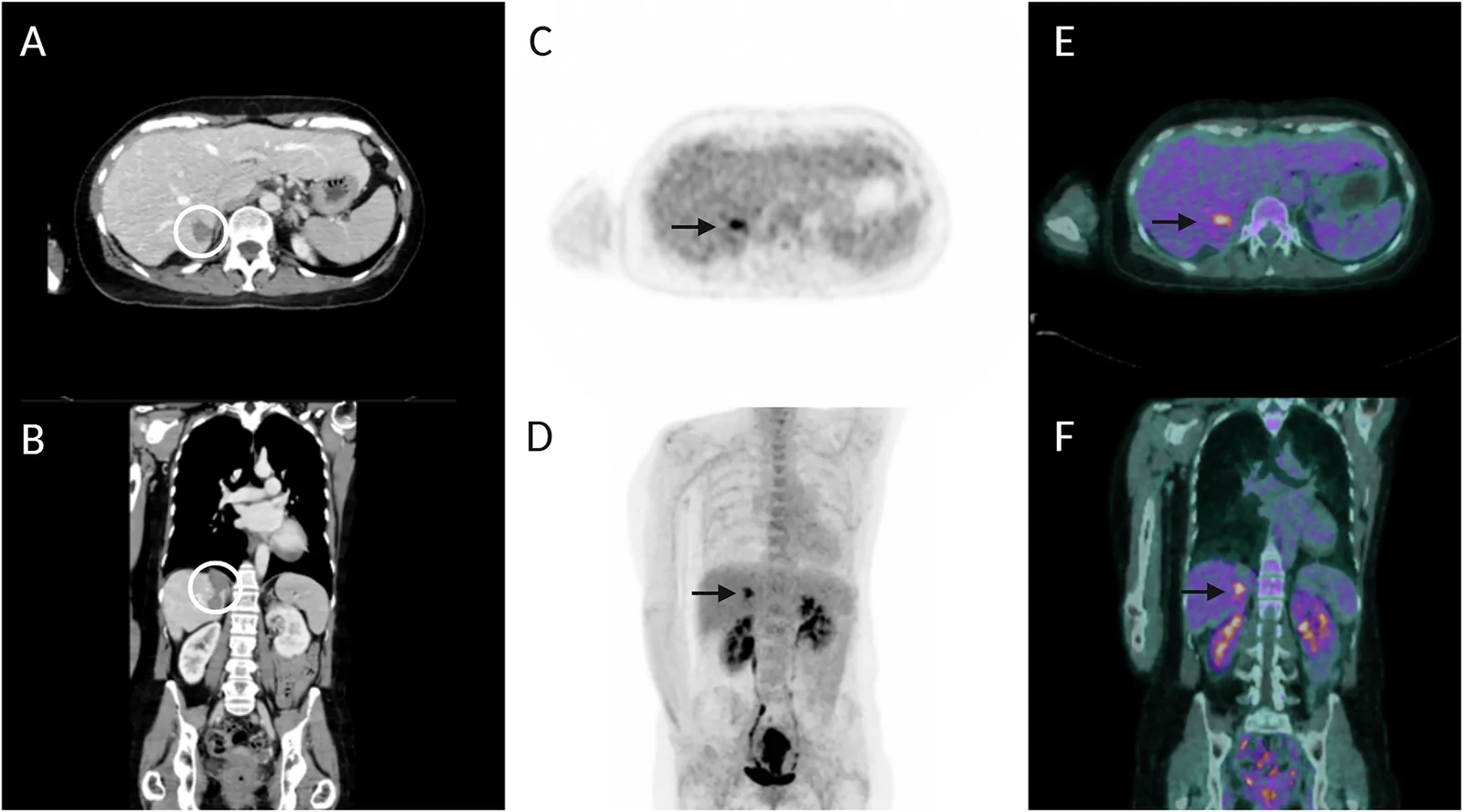
Follow-up imaging with CECT (**A**, **B**) and [^18^F]FDG-PET/CECT (**C**–**F**) three months after thermal ablation. Visible are typical postinterventional features, i.e., well-defined hypodense area at the ablation zone without focal enhancement along the border or irregularity of the border on the CECT-images, but also a focal area of high [^18^F]FDG-uptake, highly suspicious for LTP only identified on [^18^F]FDG-PET/CT (arrows) and not on CECT (circles). Please note the importance of CECT combined with [^18^F]FDG-PET to correlate metabolic and morphologic features

**Fig. 2:**
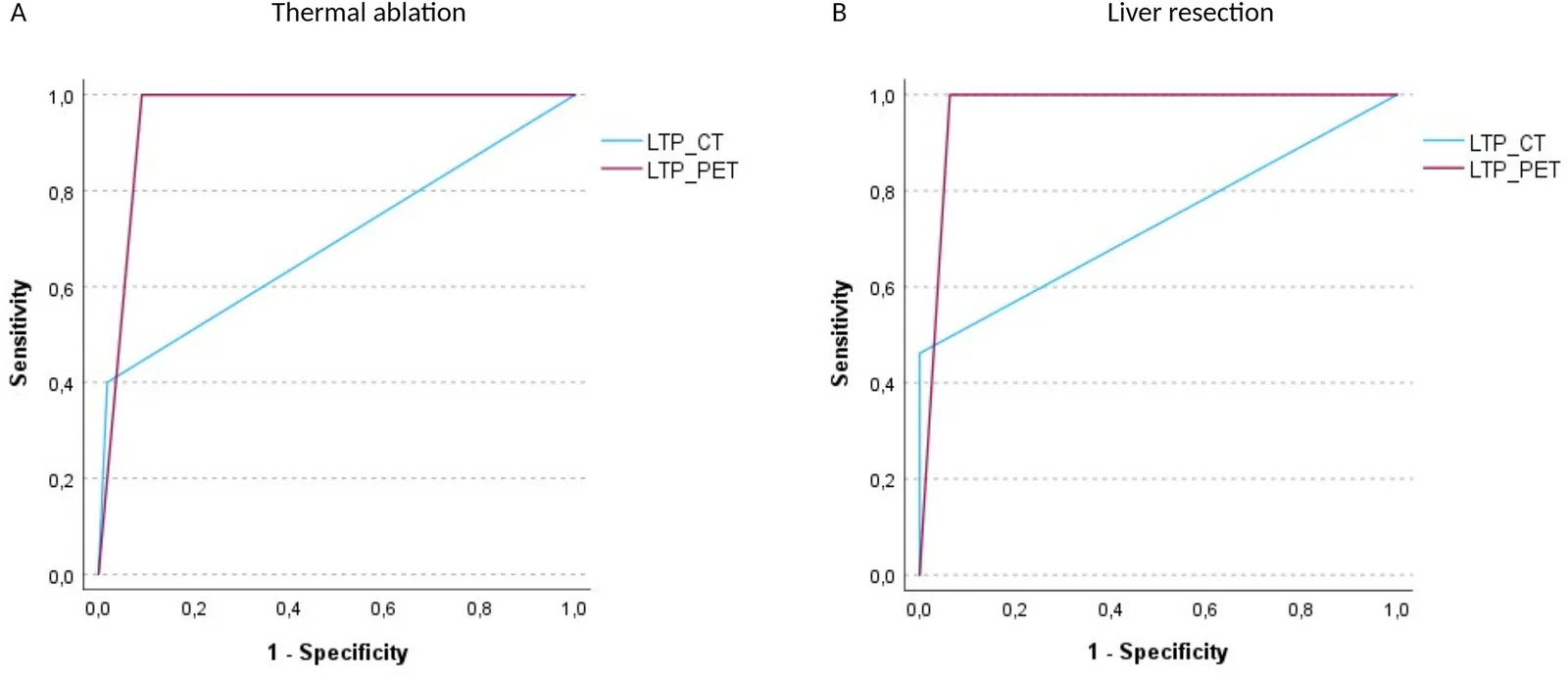
**A** Receiver operating characteristic (ROC)-curves of the diagnostic accuracy of CECT (blue) and [^18^F]FDG-PET/CECT (red) for the detection of LTP after thermal ablation. **B** ROC-curves of the diagnostic accuracy of CECT (blue) and [^18^F]FDG-PET/CECT (red) for the detection of LTP after hepatic resection

LTP was found in 13 of 93 (14%) lesions treated by thermal ablation at first follow-up. CECT correctly identified 6 of 13 (46%) LTPs and all (80) treated lesions without progression. Sensitivity and specificity of CECT for LTP were 46% and 100%, respectively, and PPV and NPV were 100% and 92%, respectively. [^18^F]FDG-PET/CECT correctly identified all (13) LTPs and was false positive in 5 out of 80 (6.3%) lesions without LTP. Sensitivity and specificity of [^18^F]FDG-PET/CECT for LTP were 100% and 94%, respectively, and PPV and NPV were 72% and 100%. The AUC for the detection of LTP after thermal ablation for CECT was significantly lower compared to [^18^F]FDG-PET/CECT (0.73 vs. 0.97, *p* = 0.001, Table 2). An example of LTP identified with [^18^F]FDG-PET/CECT imaging, which was not seen on CECT alone, is provided in Fig. 1. ROC-curves of the diagnostic accuracy of CECT and [^18^F]FDG-PET/CECT for the detection of LTP after thermal ablation are displayed in Fig. 2A.

**Table 2:** Diagnostic accuracy measures for CECT and [^18^F]FDG-PET/CECT imaging for LTP detection after thermal ablation and hepatic resection

	Thermal ablation (*n*=93 lesions)		Hepatic resection (*n*=61 lesions)	
	CECT	[^18^F]FDG-PET/CECT	CECT	[^18^F]FDG-PET/CECT
Sensitivity	46%	100%	40%	100%
Specificity	100%	94%	98%	91%
PPV	100%	72%	67%	50%
NPV	92%	100%	95%	100%
AUC	0.73	0.97	0.69	0.96

### Diagnostic accuracy of CECT and [^18^F]FDG-PET/CECT for the detection of LTP after liver resection

LTP was found in 5 of 61 (8%) lesions treated by hepatic resection at first follow-up. CECT correctly identified 2 of 5 (40%) LTPs and correctly diagnosed 55 out of 56 (98%) lesions without LTP. Sensitivity and specificity of CECT for LTP were 40% and 98%, respectively, and PPV and NPV were 67% and 95%, respectively. [^18^F]FDG-PET/CECT correctly identified all LTPs and showed no false negative lesions. Sensitivity and specificity were 100% and 91%, respectively, and PPV and NPV were 50% and 100%, respectively. The AUC for the detection of LTP after hepatic resection for CECT was significantly lower compared to [^18^F]FDG-PET/CECT (0.69 vs. 0.96, *p* = 0.03, Table 2). ROC-curves of the diagnostic accuracy of CECT and [^18^F]FDG-PET/CECT for the detection of LTP after hepatic resection are displayed in Fig. 2B.

### Diagnostic accuracy of CECT and [^18^F]FDG-PET/CECT for the detection of new CRLM and extrahepatic disease

*S*ixteen patients (25%) were diagnosed with 28 new CRLM at the time of first follow-up imaging with a mean lesion size of 10.6 mm (standard deviation 4.2 mm). CECT and [^18^F]FDG-PET/CECT correctly identified new CRLMs in 12 and 13 patients, respectively, and were false positive in 5 and 3 patients, respectively. Diagnostic accuracy of CECT and [^18^F]FDG-PET/CECT for the detection of new CRLM was comparable (AUC 0.82 vs. 0.87, respectively, *p* = 0.17, Table 3).

**Table 3:** Diagnostic accuracy measures for CECT and [^18^F]FDG-PET/CECT for the detection of new CRLM and extrahepatic disease

	Detection of new CRLM		Detection of EHD	
	CECT	[^18^F]FDG-PET-CT	CECT	[^18^F]FDG-PET-CT
Sensitivity	75%	81%	72%	86%
Specificity	90%	94%	83%	95%
PPV	71%	81%	70%	90%
NPV	91%	94%	85%	93%
AUC	0.82	0.87	0.79	0.91

Twenty-two (34%) patients had EHD at first follow-up imaging. CECT correctly identified extrahepatic disease in 16 (72%) of these 22 patients, and [^18^F]FDG-PET/CECT correctly identified EHD in 19 (86%) patients. CECT and [^18^F]FDG-PET/CECT were false positive in 7 and 2 patients, respectively. Diagnostic accuracy of [^18^F]FDG-PET/CECT was higher than that of CECT for the detection of EHD (AUC 0.91 vs. 0.79, respectively, *p* = 0.03, Table 3). ROC-curves of the diagnostic accuracy of CECT and [^18^F]FDG-PET/CECT for the detection of new CRLM and EHD are displayed in Supplementary Figs. 2 and 3.

### The value of CEA for the detection of disease progression

At diagnosis of CRLM, 43 (67%) patients had elevated CEA serum levels. A total of 42 (66%) patients showed disease progression at any site at the first follow-up. Elevated CEA serum levels (≥ 3.8 μg/mL) were found in 23 (36%) patients at the time of first follow-up. CEA serum level was false negative in 18 of 42 (43%) patients diagnosed with any progression of disease. CEA serum level was false positive in 5 cases (8%). CEA serum level as a standalone marker had a sensitivity and specificity of 43% and 77%, respectively. PPV and NPV were 78% and 41%, respectively, with an AUC of 0.60. In the subgroup of patients with elevated CEA serum levels before local treatment, the diagnostic accuracy of CEA serum levels was similar for the detection of disease progression, as sensitivity and specificity were 58% and 71%, respectively, PPV and NPV were 75% and 53%, respectively, and AUC was 0.64 (*p* = 0.57).

## Discussion

We evaluated the value of adding [^18^F]FDG-PET/CT to standard practice consisting of CECT and serum CEA 3–5 months after local treatment of CRLM in 64 patients. Our data showed that combined [^18^F]FDG-PET/CECT more accurately detects LTP compared to standard practice alone after thermal ablation (*p* = 0.001) and hepatic resection (*p* = 0.03). Combined [^18^F]FDG-PET/CECT was also more accurate in detecting EHD compared to CECT alone (*p* = 0.03). The diagnostic accuracy for the detection of new CRLM was comparable for CECT and [^18^F]FDG-PET/CECT. Moreover, we showed that measuring CEA serum levels alone does not provide sufficient information on disease control or progression. When CEA serum levels are increased, patients are more likely to have disease progression. However, low CEA serum levels should be interpreted with caution, as they result in false negatives in almost half of patients with disease recurrence.

Our results are in line with previously published studies in which [^18^F]FDG-PET/CT has been shown to be able to detect LTP after thermal ablation of CRLM [10, 15–19]. However, these studies did not directly compare [^18^F]FDG-PET/CECT to CECT, only used [^18^F]FDG-PET/low-dose CT scans or had small sample sizes. More importantly, no studies have been performed that show superiority of [^18^F]FDG-PET/CECT over CECT for the detection of residual disease or LTP after liver resection. Superiority is mainly attributed to the minimization of false negative test results without compromising false positives [8, 20]. Data on the value of [^18^F]FDG-PET/CECT for the detection of new CRLM and EHD after local treatment of CRLM were still limited until now. However, CECT has been shown to be superior to [^18^F]FDG-PET/CT for the detection of CRLM in patients without a medical history of CRLM [21, 22]. No difference in the diagnostic accuracy between CECT and [^18^F]FDG-PET/CECT for the detection of new CRLM was found in this cohort, which could be attributed to the small group sizes.

Our data showed recurrent disease at any site (i.e., LTP, new CRLM, and EHD) in 66% of patients at first follow-up, and LTP in a quarter of patients. These numbers are comparable to findings in previous studies [23–25]. The occurrence of LTP after thermal ablation was comparable to LTP after hepatic resection (14% vs. 8%, *p* = 0.3). Recently, the non-inferiority of thermal ablation compared to hepatic resection was demonstrated for small (≤ 3 cm) lesions in the multicenter randomized phase III COLLISION trial [26], which is in line with our results.

This study has several limitations. First, selection bias could have occurred when selecting patients for either of the two local treatment strategies, e.g., patient age, American Society of Anesthesiologists (ASA) classification or recurrent CRLM. However, after the hospital’s imaging protocol was initiated, all consecutive patients were prospectively identified and included in the current analysis. Second, the size of the treatment groups differed, and all groups were relatively small and heterogeneous. Nonetheless, we were able to find marked differences in diagnostic accuracy between CECT and [^18^F]FDG-PET/CECT, which should be confirmed by a future prospective trial. Third, all CECT and [^18^F]FDG-PET/CECT scans were evaluated by two abdominal radiologists and nuclear medicine physicians, respectively. In case of disagreement, a consensus was reached. Our data depicts that consensus was more often (62%) found in marking a lesion suspicious than benign, and disagreement was more frequently found when evaluating CECT scans than [^18^F]FDG-PET/CECT (16% vs. 9%). Therefore, we argue that our study is underestimating the number of false-negative results of CECT scans compared to what would be expected in the daily clinical practice when no double reading is performed. The number of false negative lesions on [^18^F]FDG-PET/CECT is probably more comparable to daily practice because less disagreement was found between the two nuclear medicine physicians. Finally, since it is hard to implement standard biopsy, the reference standard was the consensus reached during the MDT meetings in most cases, which could result in inaccurate conclusions, which further substantiates the need for a well-structured prospective study. However, pathological confirmation after additional local treatment and the follow-up period of up to 3 years confirmed the conclusions of the MDT meetings in nearly all cases (95%).

## Conclusion

Adding [^18^F]FDG-PET/CT to standard care (CECT and CEA serum level monitoring) improved the detection of LTP after thermal ablation and liver resection and was more accurate for identifying EHD. However, [^18^F]FDG-PET/CECT offered no added benefit for identifying new CRLM. CEA serum levels had low diagnostic accuracy in detecting disease progression. Integrating [^18^F]FDG-PET/CECT after local treatment of CRLM could enhance treatment decision-making. Further research should explore its impact on patient outcomes and healthcare costs.

## Supplementary information

ELECTRONIC SUPPLEMENTARY MATERIAL

## Abbreviations

18F-FDG: 2-deoxy-2-[^18^F]fluoro-D-glucose
AUC: Area under the curve
ASA: American Society of Anesthesiologists
CEA: Carcinoembryonic antigen
CECT: Contrast-enhanced computed tomography
CRC: Colorectal cancer
CRLM: Colorectal liver metastases
CT: Computed tomography
EANM: European Association of Nuclear Medicine
EARL: EANM Research Ltd
EHD: Extrahepatic disease
ESMO: European Society for Medical Oncology
LTP: Local tumor progression
MDT: Multidisciplinary team
MRI: Magnetic resonance imaging
MWA: Microwave ablation
NPV: Negative predictive value
PET: Positron emission tomography
PPV: Positive predictive value
RFA: Radiofrequency ablation
ROC: Receiver operating characteristic
US: Ultrasound
