# Supplementary material for: Improved detection of local tumor progression after ablation or resection of colorectal liver metastases using [18F]FDG-PET/contrast-enhanced CT versus contrast-enhanced CT alone: results from a Dutch prospective cohort
Source: Eur Radiol. 2026 Jul 11;36(11):8197–205. doi: 10.1007/s00330-026-12726-x (PMC13661756; doi:10.1007/s00330-026-12726-x)
Supplement: Supplementary file 1 [file 330_2026_12726_MOESM1_ESM.pdf]

Improved detection of local tumor progression after ablation or resection of colorectal liver metastases using [18F]FDG-PET/contrast-enhanced CT versus contrast-enhanced CT alone: Results from a Dutch prospective cohort

ELECTRONIC SUPPLEMENTARY MATERIAL

Eur Radiol (2026) Bijlstra, OD; van Mossel, S; Boekestijn, B et al.

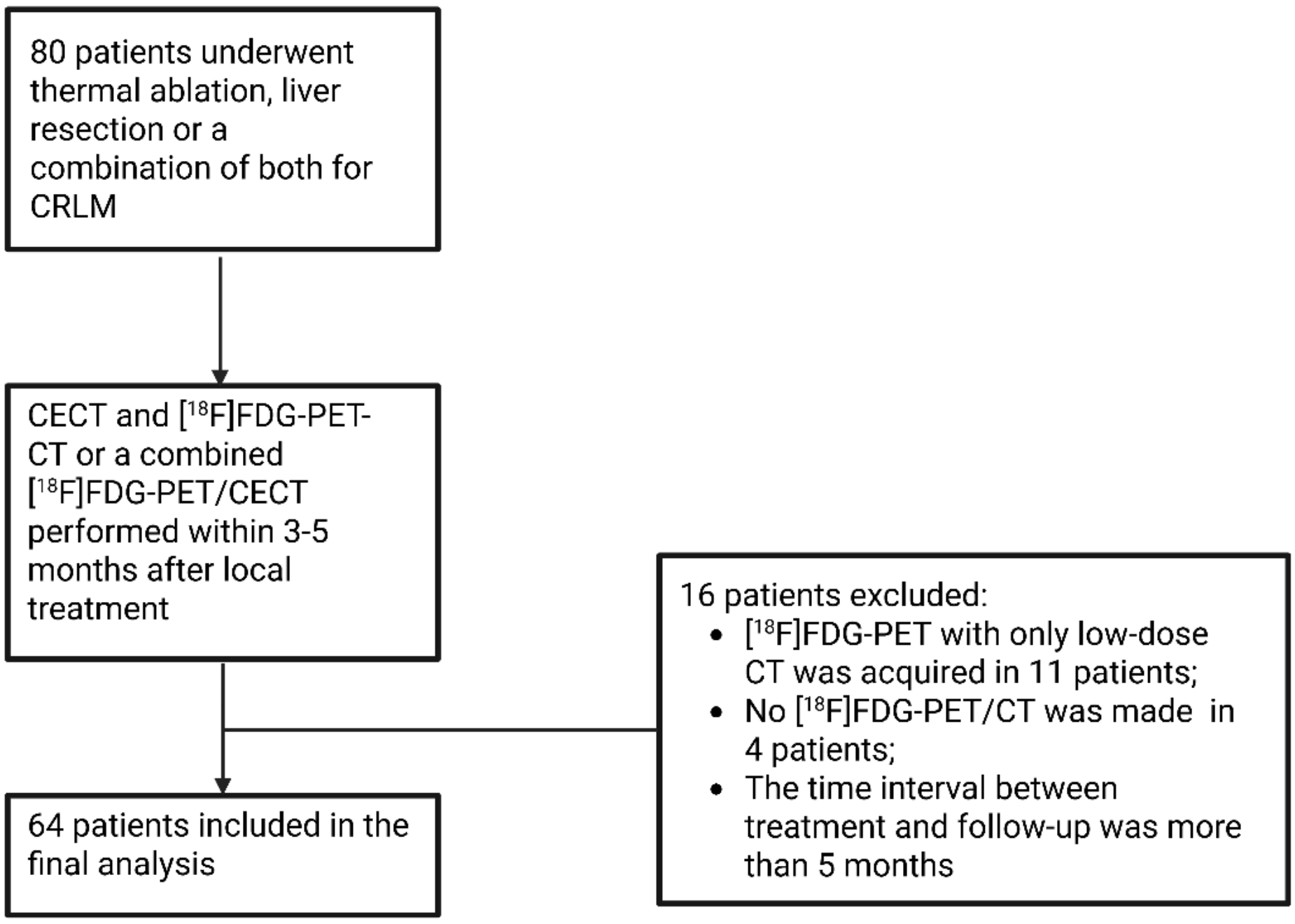

Supplementary figure 1. Patient screening flow chart.

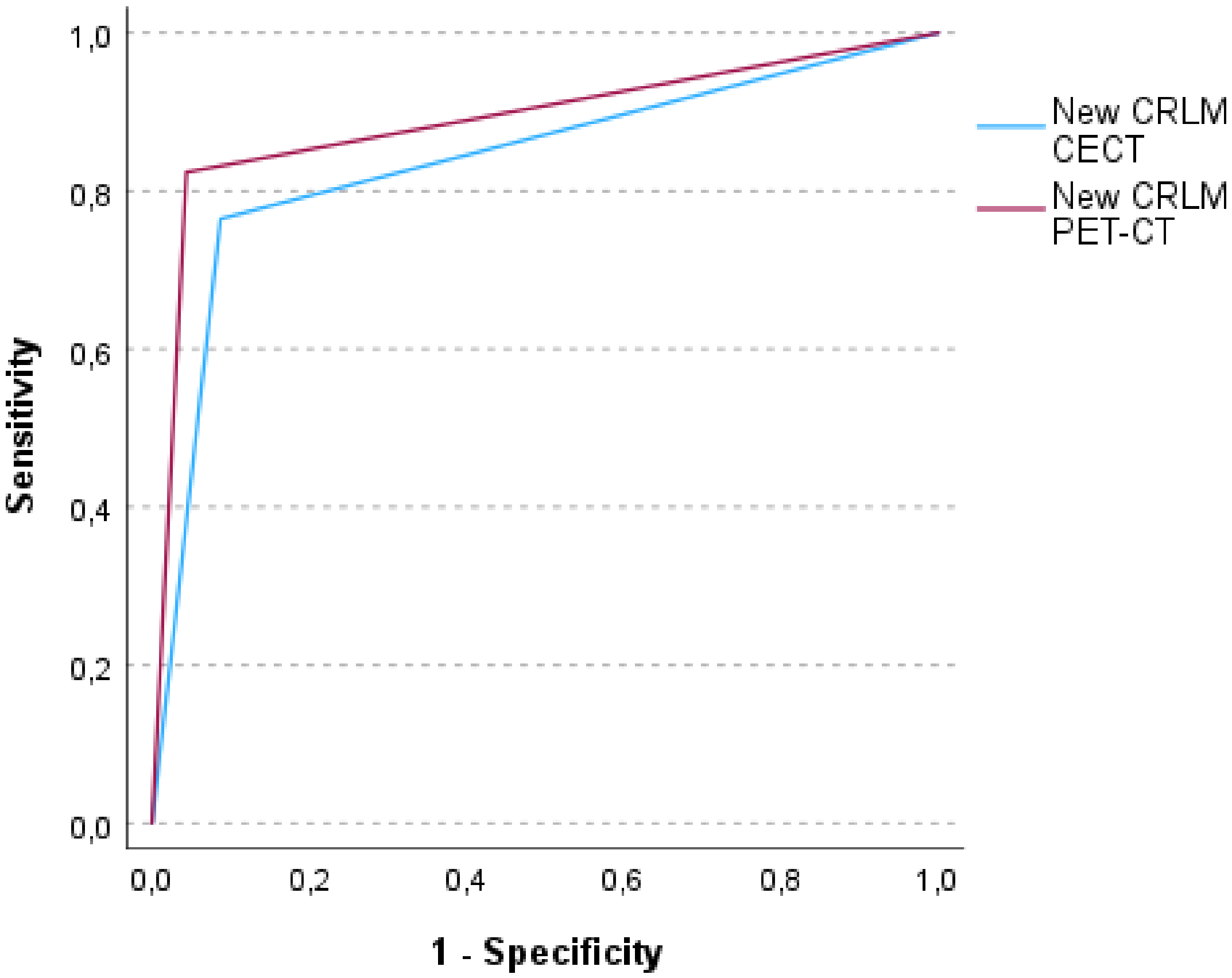

Supplementary figure 2. ROC-curves of the diagnostic accuracy of CECT (blue) and [^{18}F]FDG-PET/CECT (red) for the detection of new colorectal liver metastases (CRLM).

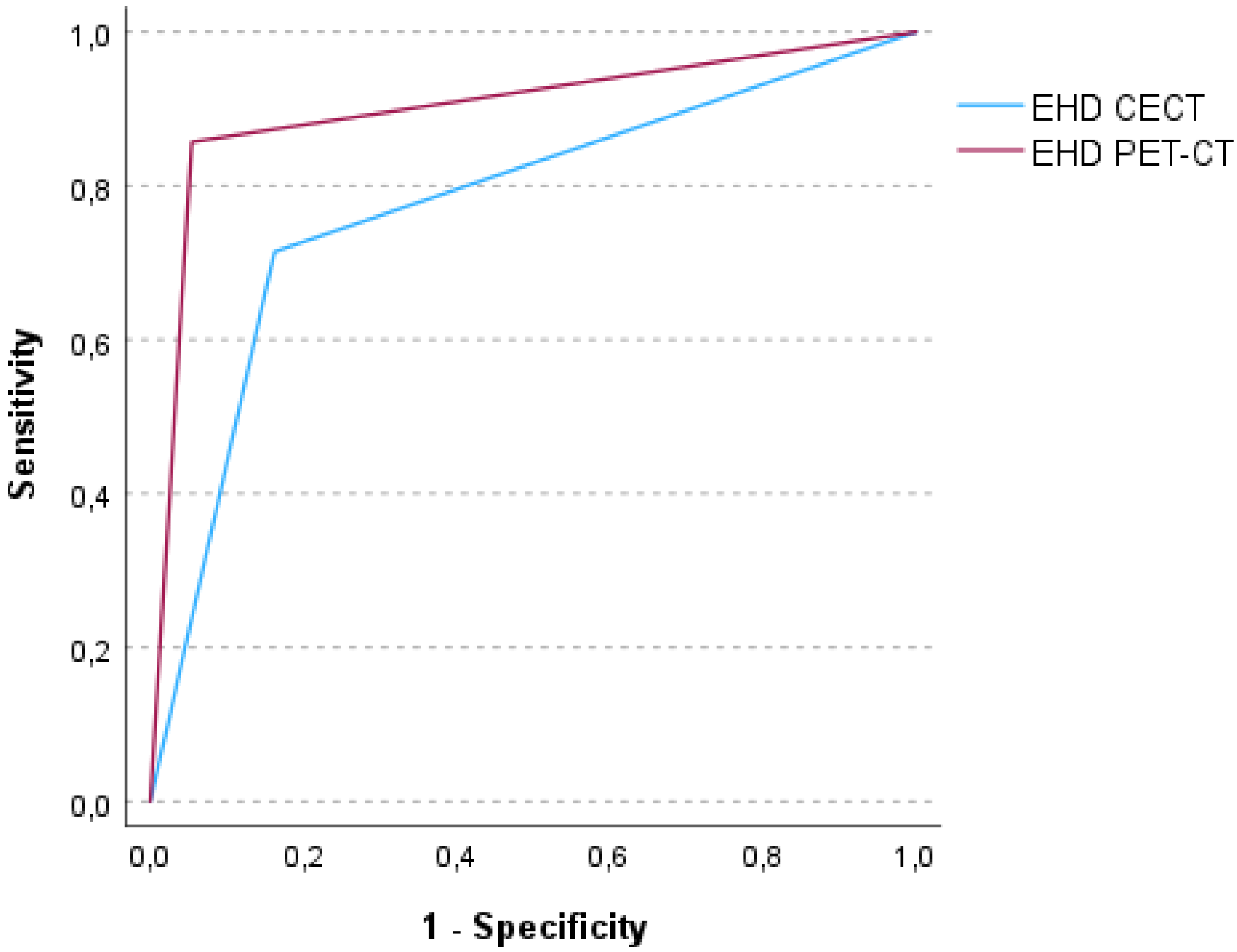

Supplementary figure 3. ROC-curves of the diagnostic accuracy of CECT (blue) and [^{18}F]FDG-PET/CECT (red) for the detection of extrahepatic disease (EHD).
